# Effect of cigarette smoke on aesthetic brackets: an in vitro study

**DOI:** 10.1590/2177-6709.27.4.e2220365.oar

**Published:** 2022-09-05

**Authors:** Laura BORGES, Amanda Cunha Regal de CASTRO, Carlos Nelson ELIAS, Margareth Maria Gomes de SOUZA

**Affiliations:** 1 Universidade Federal do Rio de Janeiro, Faculdade de Odontologia, Departamento de Ortodontia e Odontopediatria (Rio de Janeiro/RJ, Brazil).; 2 Instituto Militar de Engenharia, Laboratório de Biomateriais (Rio de Janeiro/RJ, Brazil).; 3 Universidade de São Paulo, Faculdade de Odontologia de Ribeirão Preto (Ribeirão Preto/SP, Brazil).

**Keywords:** Orthodontic brackets, Tobacco products, Smoke, Shear strength

## Abstract

**Objective:**

The objective of this study was to evaluate the effect of cigarette smoke (CS) on physical and mechanical properties of ceramic, polycarbonate and alumina ceramic brackets. The null hypothesis tested was that aesthetic brackets would not be influenced by CS.

**Methods:**

Ninety aesthetic brackets were allocated to three groups (n = 30): ceramic (GCE), polycarbonate (GCO) and alumina ceramic (GPS). Ten samples of each group were assigned to color and surface roughness analysis, performed before (T_0_) and after (T_1_) exposure to CS; and twenty samples were allocated into control and experimental groups (n = 10) (not exposed and exposed to CS, respectively) for shear bond strength test (SBS). Exposure to CS followed an adaptation of the method described by Le Mesurier. Colorimetric reading, surface morphology and roughness, SBS and adhesive remnant index (ARI) were assessed. Statistical analysis comprised independent and paired *t*-tests, ANOVA/Tukey and Fisher’s exact tests (α = 0.05).

**Results:**

Changes were observed in brackets’ color (NBS: GCE = 2.4; GCO = 1.9; GPS = 2.1), surface roughness (ΔRa: GCE = 1.1 ± 0.8 µm; GCO = 1.9 ± 1.5µm; GPS = -0.3 ± 0.1 µm / ΔRz: GCE = 1.4 ± 1.0 nm; GPS = -0.5 ± 0.1 nm); and SBS (GPS - experimental = 221.8 ± 48.6 N) after exposure to CS (*p*< 0.05).

**Conclusions:**

Exposure, *in vitro*, of aesthetic brackets to CS resulted in changes of color to darker and more opaque shades, surface roughness alterations, and higher SBS values. ARI scores were not associated with exposure to CS.

## RESUMO

**Objetivo:**

O objetivo do presente estudo foi avaliar os efeitos da fumaça do cigarro (FC) nas propriedades físicas e mecânicas de braquetes cerâmicos, de policarbonato e de cerâmica de alumina. A hipótese nula testada foi de que os braquetes estéticos não seriam influenciados pela FC.

**Métodos:**

Noventa braquetes estéticos foram alocados em três grupos (n = 30): cerâmica (GCE), policarbonato (GCO) e cerâmica de alumina (GPS). Dez amostras de cada grupo foram designadas para análise de cor e rugosidade superficial, realizada antes (T_0_) e após (T_1_) exposição à FC, e vinte amostras foram alocadas nos grupos controle e experimental (n = 10) (não expostos e expostos à FC, respectivamente) para o teste de resistência ao cisalhamento (RC). A exposição à FC foi realizada de acordo com o método descrito por Le Mesurier. Leitura colorimétrica, morfologia e rugosidade de superfície, RC e índice de adesivo remanescente (ARI) foram avaliados. A análise estatística foi realizada por meio dos testes *t* independente e para amostras pareadas, ANOVA/Tukey e teste exato de Fisher (α = 0,05).

**Resultados:**

Foram observadas alterações na cor (NBS: GCE = 2,4; GCO = 1,9; GPS = 2,1), rugosidade superficial (ΔRa: GCE = 1,1 ± 0,8 µm; GCO = 1,9 ± 1,5 µm; GPS = -0,3 ± 0,1 µm / ΔRz: GCE = 1,4 ± 1,0 nm; GPS = -0,5 ± 0,1 nm) e RC (GPS - experimental = 221,8 ± 48,6 N) dos braquetes após exposição à FC (*p*< 0,05).

**Conclusões:**

A exposição *in vitro* de braquetes estéticos à FC resultou em mudanças na cor, para tons mais escuros e mais opacos; alterações na rugosidade superficial e maiores valores de RC. Os resultados do ARI não foram associados à exposição à FC.

## INTRODUCTION

Orthodontics aims to achieve functional efficiency of the stomatognathic system, facial and dental aesthetics, periodontal health, as well as the stability of treatment outcomes. Among these, the search for aesthetic improvement is one of the main reasons why patients seek orthodontic treatment.^1^

Aesthetic brackets may be translucent, made of plastic or monocrystalline ceramic (called sapphire); or non-translucent, made of plastic or polycrystalline ceramic (machined or injected). However, the aesthetic property is also directly related to good color stability.^2^

Monocrystalline and polycrystalline ceramic brackets have been reported to resist staining or discoloration caused by substances that can be found in the mouth,^3^ and that plastic brackets have undesirable effects of darkening (staining) after a short time in the oral cavity.^4^ However, ceramic and plastic brackets may be affected by endogenous and exogenous factors when exposed to the oral environment.^5^

As exogenous factors, the aesthetic brackets are susceptible to changes in its optical properties due to pigment staining in food and beverages.^6^ However, the endogenous discoloration might be caused by UV irradiation and thermal energy.^4^

The effect of aging and chemical agents on the color stability of aesthetic brackets may vary according to their constitution, morphology, and surface characteristics, and may influence their aesthetic performance.^3^ ^,^ ^7^

In the case of smoking patients, the oral cavity is susceptible to cigarette smoke, which is constituted of toxic substances, as carbon monoxide, ammonia, nickel, arsenic, tar, lead and cadmium.^8^

According to the World Health Organization (WHO), there are 1.1 billion adult smokers worldwide, a number that has remained virtually unchanged since 2000. Brazil ranks eighth in the absolute number of smokers, about eleven million men and seven million women.^9^

In the oral cavity, the cigarette smoke compounds impregnates tooth and resin composites surfaces , incorporating yellow and blackish pigments.^10^ ^,^ ^11^

Thus, it is also questioned whether aesthetic brackets may suffer color changes from exposure to cigarette smoke, and the heat generated by smoking. Therefore, this study aimed to analyze the color change, surface roughness and resistance to debonding of aesthetic brackets when exposed to cigarette smoke. The null hypothesis of this study was that physical and mechanical properties of aesthetic brackets would not be influenced by cigarette smoke.

## MATERIAL AND METHODS

### ETHICAL CONSIDERATIONS

This research was approved by the *Comissão de Ética no Uso de Animais* (CEUA) at the *Centro de Ciências da Saúde* of the *Universidade Federal do Rio de Janeiro*, registered with the *Conselho Nacional de Controle de Experimentação* (CONCEA) (protocol number: 01200.001568. / 2013-87).

A previous sample calculation, as described by Pandis,^12^ considering an alpha of 0.01 and power of 90%; required a minimum of 9.3 samples, in order to detect a color change of 1 in ΔE measurement, with a standard deviation of 0.56 mm.^13^ Then, a minimum of 10 samples for each group was considered in the present study. Ninety aesthetic brackets were allocated to three groups (n=30) according to the type of bracket material: GCE - ceramic brackets (Ceramic); GCO - polycarbonate brackets (Composite); and GPS - ceramic of alumina brackets (PolySafira) (Morelli^®^, Sorocaba/SP, Brazil). Samples from each group were further divided according to experimental tests, as follows: 10 samples were assigned to color and roughness analysis, evaluated before (T_0_) and after (T_1_) exposure to cigarette smoke; the remaining 20 samples from each group were allocated to the shear bond strength test, of which 10 samples were not exposed to cigarette smoke (control group) and 10 samples were exposed to cigarette smoke (experimental group).

**Figure 1:**
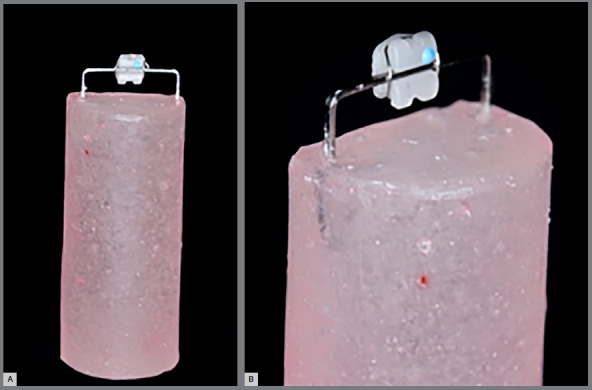
Photograph of specimens produced with steel wire **(**A); bracket tied to the specimen wire **(**B).

To perform the analyzes of color and surface roughness changes, the brackets were tied to a 0.018 x 0.025-in rectangular steel wire (Eurodonto, Curitiba/PR, Brazil), with the aid of a 0.20-mm steel wire (Morelli, Sorocaba/SP, Brazil), which was included in a test specimen, made with self-curing acrylic resin (JET - Classic, Campo Limpo Paulista/SP, Brazil) (Fig 1).

For the shear bond strength test, 60 bovine incisors crowns were included in PVC tubes of 32 mm (diameter) x 25 mm (height). The buccal surface of each crown was positioned parallel to the ground over a glass plate previously isolated with vaseline (Beira Alta, São Paulo/SP, Brazil). Then, PVC tubes were filled with self-curing acrylic resin (JET - Clássico, Campo Limpo Paulista/SP, Brazil) and the bonding area was determined on the center of the crowns’ buccal surface. 

### BONDING PROTOCOL

Bracket bonding was performed for the three groups studied (GCE, GCO and GPS), according to the protocol recommended by the manufacturer: prophylaxis of the bonding surface, followed by acid conditioning for 30 seconds with 37% phosphoric acid, then washed and dried with the triple syringe. Orthoprimer (Morelli, Sorocaba/SP, Brazil) was applied on the enamel surface, followed by air jet and light-curing for 10 seconds (Emitter G Schuster, Santa Maria/RS, Brazil). The Orthoprimer was also applied at the base of the bracket, and followed by a small amount of Orthobond Plus resin (Morelli, Sorocaba/SP, Brazil). The bracket was positioned in the delimited area, the excessive adhesive was removed and light-cured during 40 seconds, 10 seconds on each proximal face of the bracket.

### EXPOSURE TO CIGARETTE SMOKE

**Figure 2:**
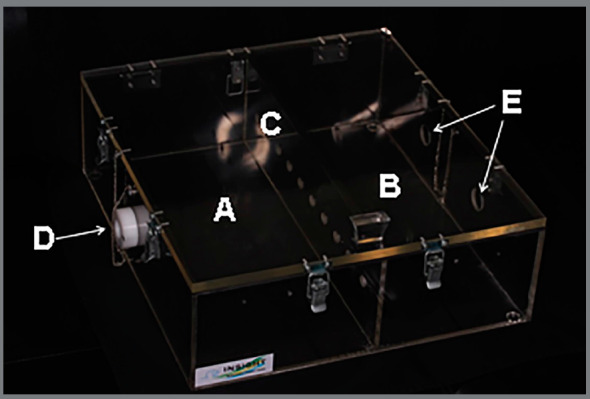
Acrylic device used for bracket’s exposure to cigarette smoke.

The method used for brackets exposure to cigarette smoke followed an adaption of Le Mesurier et al.^14^ The exposure was performed in a hermetically sealed acrylic device, that contained two chambers (Fig 2A and 2B), separated by a partition containing ten holes (Fig 2C), an air inlet (Fig 2D), and two air outlet holes (Fig 2E).

**Figure 3:**
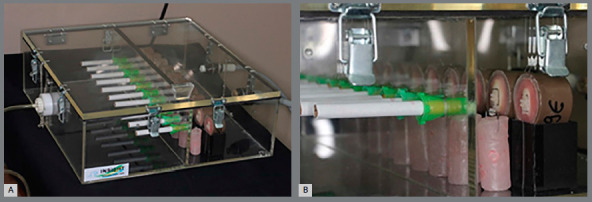
Photograph of the acrylic device for exposure to smoke **(**A); Cigarettes and specimens positioned inside the acrylic device for exposure to smoke **(**B).

The cigarettes were positioned in the holes of the partition with the aid of plastic supports, so that the filter was facing the camera B, where the specimens were positioned. After the cigarettes were turned on and the device was capped, camera A received external ventilation from an ultrasonic inhaler (US-800 air, ICEL, São Paulo/SP, Brazil) coupled to port D to provide constant airflow; and in chamber B, two suckers were coupled into holes E to create a negative pressure and cause air to pass through the cigarette filter barrier so that the smoke came into contact with the specimens (Fig 3).

Each specimen was exposed to the smoke of 20 cigarettes (Wiston, JTI, Santa Cruz do Sul/RS, Brazil), and each cigarette burned in an average time of 4 minutes. For each 5 cigarettes that were burned, the specimens were washed with water spray for 5 seconds and placed in an ultrasonic cleaning machine (Cristófolli, Campo Mourão/PR, Brazil) with distilled water for a 50 second cycle, and then dried with air jet, in order to remove the excess of the substances of the smoke that were impregnated in the surface of the brackets.

### SHEAR BOND STRENGTH TEST

The shear bond strength test was performed in a universal testing machine (EMIC DL 2000, São José dos Pinhais/PR, Brazil), with a shear load of 500 gf, at a speed of 1 mm/min. 

### ADHESIVE REMNANT INDEX (ARI) ANALYSIS

The teeth were inspected under a stereomicroscopic (model 1005t, Opticam, São Paulo/SP, Brazil) connected to a digital camera (CMOS 10 megapixels, Opticam, São Paulo/SP, Brazil). Adhesive remnant index (ARI) was scored as follows: 0 = no adhesive adhered to the enamel;1 = less than half of the adhesive adhered; 2 = more than half of the adhesive adhered; and 3 = all adhesive material adhered.^15^

### COLOR CHANGE EVALUATION

The colorimetric reading of brackets’ buccal surfaces was performed using the VITA Easyshade spectrophotometer (Model DEASYC220, Bad Säckingen, BW, Germany), by the same operator and under equal conditions of setting and exposure to artificial light, before (T_0_) and after the protocol of exposure to cigarette smoke (T_1_). Three measurements were performed for each bracket, with the spectrophotometer tip positioned perpendicular to the brackets’ buccal face with the aid of a holder. The value obtained for each specimen (L*a*b*) corresponded to the mean of these measurements. 

The color was analyzed according to the International Commission of I’Eclairage (CIE) color scale, concerning the lighting pattern D65, that distributes the color by a mathematical process of the colorimetric curve into three fields, as follows: “L” or “ΔL”, which represents brightness, or color values ​​from black to white; “a” or “Δa”, comprising colors from green to red; and “b” or “Δb”, comprising colors from yellow to blue.^16^

The color change (ΔE) was calculated by the following equation: ΔE = [(ΔL)^2^ + (Δa)^2^ + (Δb)^2^]1/2, where ΔL, Δa, Δb are the differences between values of “L”, “a” and “b” from T_0_ to T_1_. Then, ΔE* was converted to the National Bureau of Standards (NBS) scale, by the equation: NBS = ΔE* x 0.92, which is used to describe the levels of perceived color variation from visual inspection.^17^ ^-^ ^20^

### THREE-DIMENSIONAL EVALUATION OF BRACKET ROUGHNESS

Surface roughness and morphology analyses were performed according to the parameters of medium roughness (Ra) and medium depth roughness (Rz), obtained with an optical rugosimeter (Zygo NewView 7100, Zygo, Middlefield, OH, USA), by means of an interferometry technique with 20x magnification. The area scanned was of 0.085 mm² (85,000 µm²).

The roughness of the brackets was evaluated in the middle area of the slot, in two stages: initial roughness (IR), measured before exposure to cigarette smoke; and final roughness (FR), measured after exposure. The roughness variation (ΔR), determined by the equation ΔR = (FR - IR) / IR, was performed for the two parameters (Ra and Rz).

### STATISTICAL ANALYSIS

Statistical analysis was performed with SPSS v. 20 software (Statistical Package of Social Sciences, SPSS Inc., Chicago, IL, USA). Data normality was verified with Shapiro-Wilk test. Evaluation of color and roughness variables before and after exposure to smoke was performed with paired *t*-tests. Comparisons between control group (not exposed) and experimental group (exposed to cigarette smoke) of shear bond strength variable was carried out with independent *t*-tests. Intergroup comparisons of color, roughness and shear bond strength variables were performed with ANOVA/Tukey test. The adhesive remnant index was evaluated by a Fisher’s exact test. The significance level of 0.05 was adopted in all analyzes.

## RESULTS

Descriptive statistics of before and after exposure to smoke results and intergroup comparisons for L*, a*, b*, ΔE and their conversions to NBS scale parameters are presented in Table 1. Noticeable color changes were observed in all study groups, with significant differences for Composite a* values (GCO before: 2.1 ± 1.3; GCO after: 2.6 ± 1.8); and Ceramic and Polysafira b* values (GCE before: 33.0 ± 4.3; GCE after: 31.4 ± 5.0; GPS before: 31.7 ± 3.0; GPS after: 30.1 ± 2.8) (*p*< 0.05). Intergroup differences were observed for L* and a* variables before exposure to smoke (L* - GCE: 91.1 ± 2.9 and GCO: 87.7 ± 2.2 / a* - GCE: 0.6 ± 1.2 and GCO: 2.1 ± 1.3); and for a* variable after exposure to smoke (a* - GCO: 2.6 ± 1.8; GPS: 0.6 ± 1.2) (*p*< 0.05). Significant differences between GCO and GPS groups were observed for b* variable before and after exposure to smoke (*p*< 0.05). No significant differences were observed for ΔE and NBS variables among the study groups (*p*> 0.05). 

**Table 1:** Descriptive statistics (mean and standard deviation), intragroup and intergroup comparisons for the parameters L*, a*, b*, ΔE and their conversions to NBS scale (description of color change).

Groups	L*			a*			b*			ΔE	NBS
	Initial	Final	p-value	Initial	Final	p-value	Initial	Final	p-value		
Ceramic	91.1 ± 2.9^b^	91.0 ± 2.4^a^	0.869	0.6 ± 1.2^a^	0.9 ± 1.4^ab^	0.225	33.0 ± 4.3^ab^	31.4 ± 5.0^ab^	0.039*	2.6 ± 1.6^a^	2.4^a^
Composite	87.7 ± 2.2^a^	88.3 ± 3.4^a^	0.287	2.1 ± 1.3^b^	2.6 ± 1.8^b^	0.033*	35.8 ± 2.9^b^	35.6 ± 3.7^b^	0.608	2.1 ± 0.8^a^	1.9^a^
Polysafira	90.2 ± 2.7^ab^	90.1 ± 2.2^a^	0.914	0.9 ± 1.3^ab^	0.6 ± 1.2^a^	0.107	31.7 ± 3.0^a^	30.1 ± 2.8^a^	0.008*	2.3 ± 1.5^a^	2.1^a^

* Indicates statistical significance with the paired t-test (α = 0.05). Different letters indicate significant intergroup differences with ANOVA/Tukey test (α = 0.05).

**Figure 4:**
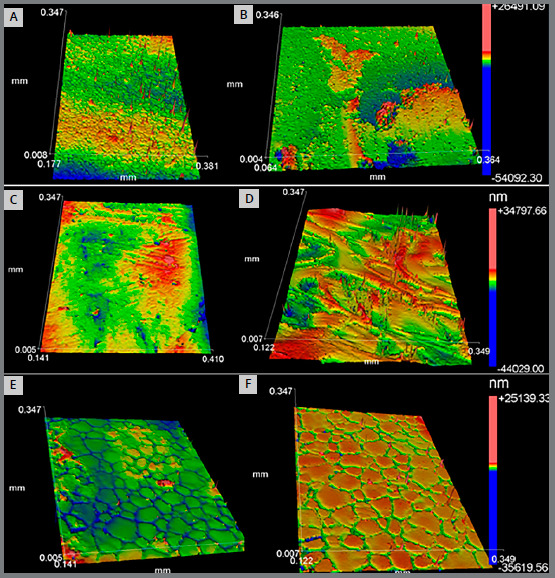
A) 3D profile of ceramic bracket before exposure to smoke. B) 3D profile of ceramic bracket after exposure to smoke. C) 3D profile of composite bracket before exposure to smoke. D) 3D profile of composite bracket after exposure to smoke. E) 3D profile of polisafira bracket before exposure to smoke. F) 3D profile of polisafira bracket after exposure to smoke.

Table 2 shows the descriptive statistics and intergroup comparisons for Ra and Rz parameters of roughness before and after exposure to smoke. Significant differences were observed in Ra parameter for all study groups before and after exposure to smoke (*p*<* *0.05). However, statistical differences for Rz parameter were only observed for Ceramic and Polysafira groups (GCE before: 24.4 ± 7.3* *nm; GCE after: 58.1 ± 23.4* *nm; GPS before: 61.0 ± 14.1* *nm; GPS after: 24.5 ± 5.6* *nm) (*p*<* *0.05). Intergroup differences were observed before and after exposure to smoke in both Ra and Rz parameters (*p*<* *0.05). Changes in bracket’s surface morphologies are illustrated in Figure 4.

**Table 2:** Descriptive statistics (mean and standard deviation), intra- and intergroup comparisons for Ra and Rz parameters of roughness.

Groups	Ra				Rz			
	Initial	Final	p-value	ΔRa (µm)	Initial	Final	p-value	ΔRz (nm)
Ceramic	0.2 ± 0.8 ^a^	0.5 ± 0.1^a^	0.003*	1.1 ± 0.8^b^	24.4 ± 7.3^a^	58.1 ± 23.4^b^	0.001*	1.4 ± 1.0^b^
Composite	0.7 ± 0.3^b^	1.7 ± 0.2^b^	0.000*	1.9 ± 1.5^b^	41.6 ± 20.3^b^	54.2 ± 3.5^b^	0.053	0.6 ± 0.8^b^
Polysafira	0.6 ± 0.1^b^	0.4 ± 0.0^a^	0.003*	-0.3 ± 0.1^a^	61.0 ± 14.1^c^	24.5 ± 5.6^a^	0.000*	-0.5 ± 0.1^a^

* Indicates statistical significance with the paired t-test (α = 0.05). Different letters indicate significant intergroup differences with ANOVA/Tukey test (α = 0.05).

Values ​​of the shear bond strength test and intergroup comparisons are presented in Table 3. Polysafira brackets presented a significant difference between control and experimental groups, as higher SBS values were observed in the group exposed to cigarette smoke (GPS - control: 165 ± 54.9* *N; experimental: 221.8 ± 48.6* *N) (*p*<* *0.05). Composite brackets presented significant decreased SBS values compared to control groups of ceramic brackets (GCO: 121.5 ± 43.3* *N; GCE: 188.3 ± 70.5* *N); and experimental groups of ceramic and polysafyra brackets (GCO: 137.3 ± 45.5* *N; GCE: 247.5 ± 77* *N; GPS: 221.8 ± 48.6* *N) (*p*<* *0.05). The highest SBS values were observed for ceramic experimental group (GCE: 247.5 ± 77.5* *N).

**Table 3:** Descriptive statistics (mean and standard deviation) and intergroup comparisons of shear bond strength (Newtons).

Groups	Control	Experimental	p-value
Ceramic	188.3 ± 70.5 ^b^	247.5 ± 77.5 ^b^	0.091
Composite	121.5 ± 43.3 ^a^	137.3 ± 45.5 ^a^	0.439
Polysafira	165.7 ± 54.9 ^ab^	221.8 ± 48.6 ^b^	0.026*

* Indicates statistical significance with independent t-test (α = 0.05). Different letters indicate significant intergroup differences with ANOVA/Tukey test (α = 0.05).

**Figure 5:**
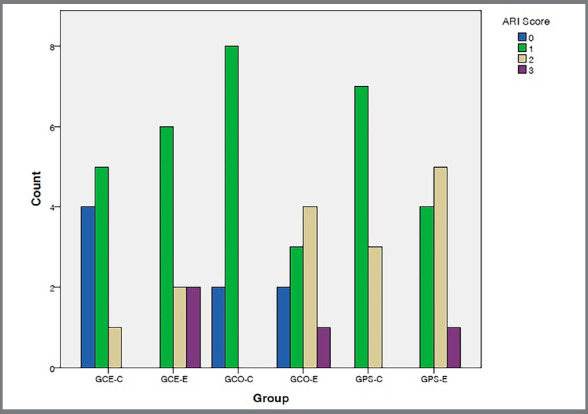
Distribution of ARI (adhesive remnant index) scores among the study groups. GCE-C: ceramic control group; GCE-E: ceramic experimental group; GCO-C: composite control group; GCO-E: composite experimental group; GPS-C: polysafira control group; GPS-E: polysafira experimental group.

The distribution of the ARI scores is shown in Figure 5. Despite the Fisher’s exact test demonstrated that there was no association of ARI scores between control and experimental groups for all types of brackets (GCE: x^2^= 5.850; *p*=* *0.121/ GCO: x^2^= 6.929; *p*= 0.051 / GPS: x^2^= 2.220; p = 0.370), it can be observed that the highest rate of ARI score 3 values was presented by the ceramic experimental group. 

## DISCUSSION

**Figure 6:**
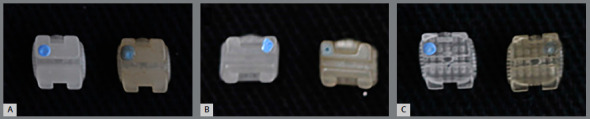
Photograph of aesthetic brackets: ceramic **(**A), composite **(**B) and polysafira**(**C) before and after exposure to smoke.

Changes in color can be detected visually or with the aid of a colorimeter. Nevertheless, the human eye is not able to observe slight color differences, and therefore, visual color interpreting and comparison is performed subjectively. Thus, in order to reproduce the results of color assessment, colorimetric measurements became necessary.^21^ The color assessment performed in the present study indicated that after exposure to smoke, brackets became visually darker and opaque (Fig 6). Despite the lack of intragroup differences for L* parameter, values ​​of b* decreased in Ceramic and Polysafira groups, and values ​​of a* increased in Composite group, after exposure to smoke. 

Ruyter et al.^22^ reported in 1987 that a color change (ΔE) of 3.3 is visually perceptible and thus clinically unacceptable. The ΔE values obtained from the present study did not exceed this limit and did not show a statistical significant difference (*p*>* *0.05) among the groups. In order to relate the value of ΔE to clinical standards, ΔE was converted to the National Bureau of Standards (NBS) scale (Table 1). The values of ΔE were converted using the equation^17^: NBS = ΔE * x 0.92, and all resulted in noticeable color changes (1.5* *<* *NBS* *<* *3.0).

Previous researches have investigated the color stability of dental materials *in vivo* and *in vitro*,^23^ ^,^ ^24^ and observed the influence of cigarette smoke in color changes and surface texture of dental composites;^13^ and also changes in color stability (ΔE > 3.3) of aesthetic brackets after exposition to UV irradiation, and to different food dyes, as red wine, coffee and tea.^4^

With the production of polycarbonate brackets in the 1970s, deficiencies such as wing fractures, distortion and staining^25^ were surpassed with ceramic reinforcement and metal slot brackets. Ceramic brackets, in general, still have a rough surface when compared to stainless steel brackets.^26^ Additionally, polycrystalline brackets have increased coefficient of friction when compared to monocrystalline ceramic and stainless steel brackets, and this is attributed to their rougher and more porous surface.^27^ In the present study, these characteristics could also be observed before exposure to smoke, as polysafira group had increased roughness values, compared to ceramic group. However, after exposure to smoke, ceramic group presented higher values ​​of roughness, in relation to polysafira group. Despite composite group had higher initial roughness (Ra) and greater roughness variation (ΔRa), it was the least affected by color variation (ΔE). In contrast, ceramic group presented lower values of initial roughness (Ra and Rz), and was the group most affected by color variation (ΔE).

An efficient ceramic bracket must provide adequate bond strength to dental enamel, but also offer easy debonding, without causing damage to tooth surface. A great advantage of ceramic brackets compared to metallic brackets is that, ceramic translucency allows the transmission of light during photopolymerization and thus, provides a higher shear bond strength^28^. According to the present results, polysafira brackets presented significant higher SBS values in specimens exposed to cigarette smoke. Composite brackets’ control group and ceramic brackets’ experimental group presented the lowest and the highest debonding values, respectively. 

Despite there was no association of ARI scores’ distribution between control and experimental groups, it is worth noting that before exposure to smoke, no case of ARI score 3 was observed, but after exposure to smoke, a certain frequency of this score was noticed. ARI score 1, in which less than half of the adhesive remains adhered to enamel surface after debonding, was predominantly frequent in the majority of the groups, which may represent more tension in the enamel-adhesive interface, and thus increase the chance of dental damage.^29^

The main limitations of this study consists of its *in vitro* design, being performed under controlled and standardized conditions. These factors may restrict the application of the present results to an oral environment, due to the influence of saliva’s flow and composition; food debris and bacterial plaque; body temperature; and tooth brushing conditions. The present findings may encourage additional studies with a longitudinal design, to further understand the influence of cigarette smoke on aesthetic brackets’ properties. 

## CONCLUSIONS

The exposure of aesthetic brackets to cigarette smoke, *in vitro*, revealed that:

» Brackets color changed to darker and more opaque shades.
» Ceramic and composite brackets presented increased average roughness (Ra) and medium depth roughness (Rz), whereas polysafira brackets presented a decrease in surface roughness parameters.
» Polysafira brackets exposed to CS presented higher SBS values, compared to non-exposed brackets.
» ARI scores were not associated to exposure to cigarette smoke.

Thus, the null hypothesis of the present study was rejected.
